# Constructing the Immune Signature of Schizophrenia for Clinical Use and Research; An Integrative Review Translating Descriptives Into Diagnostics

**DOI:** 10.3389/fpsyt.2018.00753

**Published:** 2019-01-31

**Authors:** Rune A. Kroken, Iris E. Sommer, Vidar M. Steen, Ingrid Dieset, Erik Johnsen

**Affiliations:** ^1^Psychiatric Division, Haukeland University Hospital, Bergen, Norway; ^2^Norwegian Centre for Mental Disorders Research, Haukeland University Hospital, Bergen, Norway; ^3^Department of Clinical Medicine, University of Bergen, Bergen, Norway; ^4^Department of Neuroscience and Department of Psychiatry, University Medical Center Groningen, University of Groningen, Groningen, Netherlands; ^5^Department of Biological and Medical Psychology, University of Bergen, Bergen, Norway; ^6^Department of Clinical Science, Norwegian Centre for Mental Disorders Research, KG Jebsen Centre for Psychosis Research, University of Bergen, Bergen, Norway; ^7^Dr. E. Martens Research Group of Biological Psychiatry, Department of Medical Genetics, Haukeland University Hospital, Bergen, Norway; ^8^Norwegian Centre for Mental Disorders Research, KG Jebsen Centre for Psychosis Research, Oslo University Hospital and University of Oslo, Oslo, Norway; ^9^Division of Mental Health and Addiction, Acute Psychiatric Department, Oslo University Hospital, Oslo, Norway; ^10^Faculty of Medicine, Institute of Clinical Medicine, University of Oslo, Oslo, Norway

**Keywords:** CRP-C-reactive protein, schizophrenia, inflammation, immunity, cytokine, anti-inflammatory drugs, monoclocal antibody, MRI

## Abstract

Schizophrenia is considered a syndrome comprised by several disease phenotypes, covering a range of underlying pathologies. One of these disease mechanisms seems to involve immune dysregulation and neuroinflammation. While the current dopamine receptor-blocking antipsychotic drugs decrease psychotic symptoms and prevent relapse in the majority of patients with schizophrenia, there is a huge need to explore new treatment options that target other pathophysiological pathways. Such studies should aim at identifying robust biomarkers in order to diagnose and monitor the immune biophenotype in schizophrenia and develop better selection procedures for clinical trials with anti-inflammatory and immune-modulating drugs. In this focused review, we describe available methods to assess inflammatory status and immune disturbances *in vivo*. We also outline findings of immune disturbances and signs of inflammation at cellular, protein, and brain imaging levels in patients with schizophrenia. Furthermore, we summarize the results from studies with anti-inflammatory or other immune-modulating drugs, highlighting how such studies have dealt with participant selection. Finally, we propose a strategy to construct an immune signature that may be helpful in selecting and monitoring participants in studies with immune modulating drugs and also applicable in regular clinical work.

## Introduction

Immune dysregulation in schizophrenia has been found in numerous studies comparing patients to healthy controls, and meta-analyses find that patients with schizophrenia, on a group level, show signs of a low-grade peripheral inflammation with upregulation of several proinflammatory cytokines (1–3) and C-reactive protein (CRP) (4). While the origin of these findings is not established, a major result from genome wide association studies (GWAS) has been a robust genetic association between schizophrenia and the major histocompatibility complex (MCH) locus on chromosome 6 (5). This genetic susceptibility can in part be explained by variants of complement factor 4 (C4), possibly linked to increased synaptic pruning during brain development (6). Furthermore, studies show increased risk of schizophrenia in individuals with prenatal exposure to influenza, although disputed (7), or with elevated titers of IgG antibodies to toxoplasma gondii (8), likely to work in concert with a genetic background (9). Interestingly, the pathological influence of prenatal infection may be an unspecific effect of having an inflammation response and increased cytokine levels more than a specific effect of a particular infectional agent (9). Moreover, studies of post-mortem brains of patients with schizophrenia suggest increased microglial activity (10). While these findings and others have broadened the knowledge of how immunity may influence ethio-pathological processes in schizophrenia, the advance of novel treatment algoritms for the individual patient would benefit from identification of robust immune-biomarkers for schizophrenia (11). In addition, theranostic biomarkers predicting effects of treatment with anti-inflammatory or immune-modulating drugs are needed (12) and descriptive group level findings must be translated into diagnostic assessment of the individual patient (11).

Although dopamine D2-receptor blocking antipsychotic drugs play a major role in the treatment of psychotic disorders (13), new treatment options are strongly needed, above all for the cognitive and negative symptoms of schizophrenia. D2-blockers offer symptomatic relief for delusions and hallucinations and efficient relapse prevention to a majority of users (14), but a disease-modifying effect in schizophrenia has not been found. Immune-modulating treatments might target pathological processes more proximal to the roots of the psychotic disorder than is the case for the current D2-receptor blocking drugs, and accordingly may be able to treat not only symptoms. Since 2002, there has been several pivotal studies exploring the potential effect of non-steroidal anti-inflammatory drugs (NSAIDS) (15), estrogens, statins, EPA/DHA fatty acids, davunetide, minocycline, and N-acetyl cysteine in schizophrenia (16). Furthermore, trials with monoclonal antibodies toward cytokines or cytokine receptors are emerging (17), which can specifically target one component of the immune system and may provide opportunities for precision medicine. This is a rapidly developing field (18), that now contains a range of well-established treatment options for various medical and neurological disorders, such as multiple sclerosis (MS). There is now a broad understanding that immune dysregulation may form an important part of the pathophysiology of schizophrenia and a whole range of drugs targeting specific parts of the immune system are already available. Following up on the studies with various anti-inflammatory acting drugs that have already been conducted, the stage is set for a new phase of drug studies in schizophrenia (19).

However, in order to maximize chances of showing effect in studies with immune-modulating drugs a schizophrenia inflammatory phenotype should be defined and delineated at the individual level both for research and clinical purposes. Several authors have highlighted that in most studies of inflammation in schizophrenia around 40% of the patients have some degree of inflammation (20–22). Assuming that immune dysregulation is involved in the pathoetiology of sub-groups with schizophrenia is in line with the notion that schizophrenia is a syndrome comprised by several disease phenotypes with a range of distinct underlying pathologies (23, 24). One of these disease mechanisms could be related to immunity, while others may be more influenced by compromised energy metabolism or synaptic dysfunctions (11). As several authors have noted already, we need robust biomarkers to diagnose immune dysregulation in schizophrenia and help selecting participants for trials with immune-modulating drugs. Further down the line, biomarkers are also needed in clinical settings in order to evaluate the individual patient for treatment. Promising indications of the possibilities that such a strategy represents derive from trials with immune-modulating drugs in depression. In two studies of infliximab which blocks tumor necrosis factor (TNF)-α in patients with major depression, treatment only benefited participants with CRP above a certain level (25, 26). Also, a schizophrenia trial stratifying results on degree of inflammation showed stronger treatment effects in the participants with increased inflammation (27).

Here we first review available methods to assess inflammatory status or immune disturbances. Findings of disturbances in immune cells, cytokines including mRNA, acute phase proteins, other molecular level methods and findings, and brain imaging methods will be outlined. Furthermore, we summarize the results from studies with anti-inflammatory or other immune-modulating drugs, highlighting how the studies have dealt with participant selection. Finally, we propose a strategy to construct an inflammatory signature that may be useful in selecting and monitoring participants in studies with immune modulating drugs and also applicable in the regular clinical work. We will start with a brief overview of the immune system.

## Immunity and Inflammation

**Figure 1:**
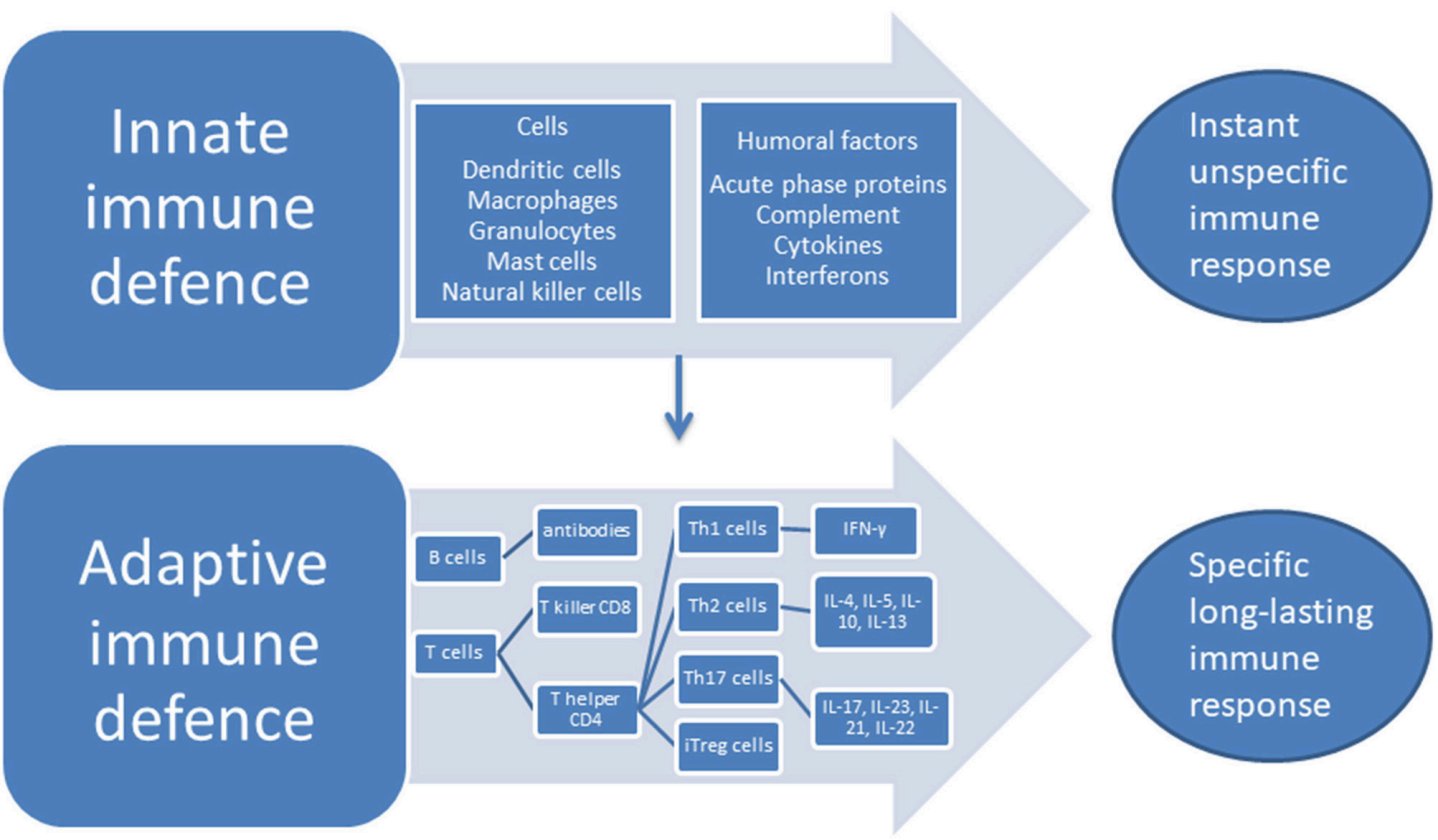
The immune system—an overview.

The two categorically different parts of the immune systems are the innate system—that responds to pathogens in an unspecific way but does not produce lasting immunity—and the adaptive system which responds to specific antigens in a way that creates long-lasting recognition. The long-lasting recognition is produced through the creation of cell lines that give a specific antibody response. The cells of the innate system are the dendritic cells (DC), the macrophages, granulocytes, mast cells, and the natural killer (NK) cells, while the humoral responses of the innate system consist of the complement system, cytokines and interferons. The cells of the adaptive system are the B and T lymphocytes, while the antibodies are the humoral part of the adaptive system (28), see Figure 1. An important part of the innate response is the toll-like receptors (TLRs) located at the macrophages where they induce phagocytosis and production of albumin, fibrinogen, and serum amyloid A protein together with CRP—the acute phase proteins (29). A further acute response consists of the production of cytokines that stimulates T and B cells into producing responses specific to the given antigen. T cells are divided into subsets on the basis of their surface receptors, and the two main types are the cluster of differentiation (CD)4 T helper(h) cell, and the CD8—the T killer cells. The CD4 Th cells secrete a major portion of the cytokines of the body. Cytokines and chemokines are small molecules that act predominantly in the microenvironment of the cells that secrete them, while interleukin (IL)-1β, transforming growth factor (TGF) and TNF are exceptions to this and can also circulate through the body (28). Cytokines and chemokines have important roles in the communication between cells in the immune system, they can have stimulatory or inhibitory effects and their role may change depending on context. When the CD4 Th cell is activated, it can differentiate into Th1 and Th2 effector cells producing different types of cytokines. Th1 cells produce interferon (IFN)- γ which has strong pro-inflammatory properties, while the Th2 cells upon stimulation produces IL-4, IL-5, IL-10, IL-13 with mixed effects (30). CD4 Th cells can also differentiate into Th17 cells and induced regulatory T (iTreg) cells. Th17 produces several cytokines with a predominantly pro-inflammatory effect, IL-17, IL-23, IL-21, IL-22, and IL-17/IL-23 induce the IL-17/IL-23 immune axis (31).

Several specific cytokines need particular attention as they are consistently reported to be associated with schizophrenia. The **IL-1 family** consists of seven proteins displaying a predominantly pro-inflammmatory function: IL-1α, IL-1β, IL-18, IL-33, IL-36a, IL-36b, IL-36g, moreover three receptor antagonists IL-1Ra, IL-36Ra, IL-38, and one cytokine with anti-inflammatory actions; IL-37 (32). The IL-1 family are pleiotropic, and also have immunoregulatory and hematopoitic effects (28). IL-1 influences antigen presentation and non-specific lymphocyte function, and is closely linked to innate immunity (33). The IL-1 receptor type 1(RI) shows strong similarities to the TLR. Binding of IL-1 can initiate and strengthen the acute phase response by inducing fever that increases migration of leucocytes, by stimulating the acute phase proteins such as CRP, by activation of the hypothalamus-pituitary-adrenal (HPA) axis with cortisol regulating innate inflammation, and by inducing adhesion molecules that increase leucocyte recruitement (32). IL1 is mainly produced by activated macrophages, which is for instance activated by interferon (IFN)-γ and bacterial products (28, 32). **IL-6 is** produced by immune cells, adipocytes, skeletal muscle cells and vascular endothelial cells, and the IL-6 receptor is located on macrophages, lymphocytes, neutrophils and hepatocytes (34). IL-6 stimulates B cell differentation and activation of T cells in acute inflammation, and promotes the synthesis of CRP, fibrinogen and albumin in the acute response (34). IL-6 influences the aforementioned differentiation of Th17 cells together with TGF-β, and constrains TGF-induced Treg cells differentiation (35). The fatigue, anorexia and fever associated with acute inflammations may be induced by IL-6 (36). However, IL6 also has a role in dampening the inflammatory response by reducing the production of IL-1β and TNF-α (37), and by inducing the production of IL-1 Ra (38) and the anti-inflammatory cytokine IL-10 (39). Recent results indicate that activation of IL-6 without a concomitant activation of IL-1β and TNF, for example during physical exercise, mostly induces anti-inflammatory actions (39). **TNF-α** is another pro-inflammatory cytokine with important functions in innate and adaptive immunity. It is produced in macrophages and monocytes, as well as in T-cells, adipocytes and smooth muscle cells and binds to tumor necrosis factor receptor type I (TNF-RI) and type II (TNF-RII). With the exception of erythrocytes, TNF-RI and TNF-RII are located on all cells of the body and are involved in pro-inflammatory pathways through the activation of nuclear factor-kB (40).

## Dysregulated Immune System and Inflammation in Schizophrenia

**Figure 2:**
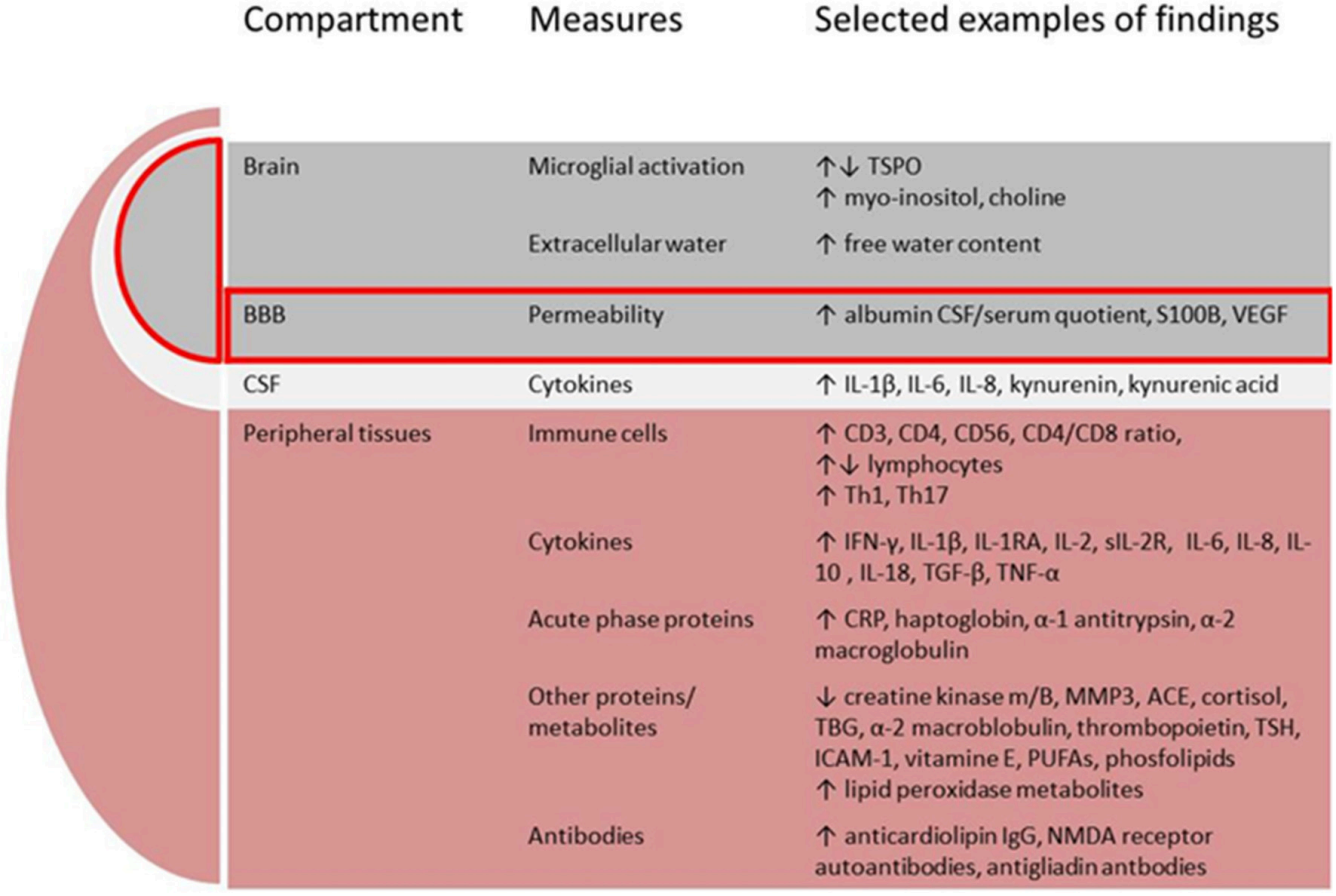
Putative components comprising the immune signature in schizophrenia. BBB, blood-brain-barrier; CSF, cerebrospinal fluid; TSPO, translocator protein; VEGF, vascular endothelial growth factor; IL, interleukin; IFN, interferon; RA, receptor antibody; R, receptor; TGF, transforming growth factor; TNF, tumor necrosis factor; MMP, matrix metalloproteinase; ACE, angiotensine converting enzyme; TBG, thyroxine-binding globuline; TSH, thyrioidea stimulating hormone; ICAM, intercellular adhesion molecule; PUFAs, poly-unsaturated fatty acids; NMDA, N-methyl-D-aspartate.

There is a growing body of evidence implicating dysregulated immunity in schizophrenia from both *in-vitro* and *in-vivo* studies. In this overview we will limit the description to studies applying tissues and methods that can potentially become useful in the clinical assessments of patients. We will present relevant immune cells, cytokines and acute phase proteins, expression of cytokine genes, other proteins and metabolites, and finally brain imaging methods used to assess neuroinflammation, see Figure 2.

### Assessments in Peripheral Tissues

#### Immune Cells

The immune cells are the cornerstones of the immune system, and it is rather unlikely that an immune disturbance of possible pathoetiological significance in schizophrenia would be present without a detectable immune cell signature. However, few studies have described immune cell disturbances so far. A meta-analysis of 16 studies of lymphocytes in schizophrenia vs. healthy controls (41) showed a significant increase in the percentage of CD4 and CD56 (natural killer cells) in acutely ill patients. Drug naïve first episode patients showed a significant increase in the levels of lymphocytes, CD3 (all T-cells) and CD4 levels, and the CD4/CD8 ratio. Absolute CD56 levels were suggested to be trait-dependent, while CD4/CD8 ratio could be state-dependent. A study of 18 patients with schizophrenia (17 treated with clozapine) vs. 18 healthy persons found elevated monocytes, NK cells, naïve B cells and CXCR5+ memory CD4 cells in the schizophrenia group, and decreased number of DC and several T cells populations. The authors find it plausible that clozapine treatment influenced the results (42). In a selective review by Bergink et al. (43) several studies report elevated monocyte counts in the periphery of patients with schizophrenia and higher gene expression for inflammatory cytokines in circulating monocytes. For circulating T cells three referred studies found reduced numbers of circulating lymphocytes, while one study found increased numbers of Th1, Th17, and suppressive natural T regulatory cells (44). In a study of 69 drug-naïve first episode patients with schizophrenia (FES) compared to 70 healthy controls, FES had significantly higher proportion of Th17 cells (45), and the proportion of Th17 cells correlated positively with PANSS total. Interestingly, after 4 weeks of treatment with risperidone, the proportion of Th17 cells decreased significantly. However, conflicting results regarding the Th17 axis have been published (30). It is clearly an advantage from a clinical point of view that immune cells can be assessed with well-established and readily available methods, for example flow-cytometry (46), and are routinely surveyed in the clinical treatment of various conditions, see for example the website of the Karolinska hospital where a full menu of lymphocyte immunphenotyping is offered (www.karolinska.se). Taken together, studies of lymphocytes as well as monocytes in patients with schizophrenia show very interesting differences compared to healthy controls, but more research is needed to evaluate immune cell counts such as lymphocyte immunophenotyping as theranostic biomarkers for immune dysregulation/inflammation in schizophrenia.

#### Cytokine Protein Levels in Serum

A major body of knowledge regarding immune dysfunction in schizophrenia derives from studies on cytokines in peripheral blood. During the last two decades, many studies have been performed and new ones are arriving (47). Others have summarized these results in systematic reviews and meta-analyses (1–3). The recent study by Rodrigues-Amorim et al. (3) also contains a very helpful summary of the function and clinical impact of the different cytokines. They included 99 studies with 8,234 participants and found that peripheral levels of the following cytokines differed between patients with schizophrenia and healthy controls in more than 50% of the included studies, listed according to falling prevalence among the studies: IL-6, TNF-α, IL-10, IFN-γ, IL-1β, IL-8, IL-2, IL-1RA, furthermore the gene polymorphisms for TNF-α 1800629, IL-6 rs1800795, and IL-1β rs16944, and elevated expression levels of IL-6, TNFR1, TNFR2, and IL-1β mRNAs (3). It is important to emphasize that the identified changes are smaller in magnitude compared to findings from for example inflammation in rheumatoid artritis and other auto-immune disorders, and collectively it is referred to as a low-grade inflammation (48).

##### Drug-naïve FES

IL-1β, soluble (s)IL-2receptor(R), IL-6, and TNF-α were significantly elevated in a meta-analysis of 23 studies with 570 subjects with drug-naïve FES vs. 683 controls (1). Also non-significant changes of IL-2, IL-4, and IFN-γ were identified. An earlier meta-analysis with 14 studies in FES (2) found IL-1β, sIL-2R, IL-6, IL-12, TNF-α, IFN-γ, TGF-β to be increased in FES vs. controls. Furthermore, in a study of 12 ultra-high risk (UHR) individuals compared to 16 healthy controls IL-17 was significantly decreased and IL-6 increased in the UHR group (49). The finding of low-grade peripheral inflammation in a subset of drug-naïve patients at the time of diagnosis is among the stronger underpinnings of the “inflammation hypothesis” in schizophrenia. However, as a general Th1/Th2 imbalance is not found the interpretation of the findings in terms of underlying immune disturbances is not clear (1).

##### Effects of antipsychotic treatment on cytokine levels

In a meta-analysis including 8 studies of drug-naïve patients with first-episode psychosis (FEP) a significant reduction after antipsychotic treatment for IL-2 and IL-6 was found. After excluding only one study IL-1β also declined significantly (50). The authors suggested that IL-1β, IL-2, and IL-6 could serve as markers for psychosis, while TNF-α, IL-17, and IFN-γ were still elevated after antipsychotic treatment. The analyses included in total between 69 (IL-2) and 253 (IL-6) subjects, and included studies with data available after 4 weeks of antipsychotic treatment. An earlier review of cytokine changes after antipsychotic treatment (4 to 52 weeks) including 39 studies with schizophrenia spectrum patients found that antipsychotic treatment was associated with reduced IL-2, increased sIL-2R and sTNF-R1/R2 and in some studies also an increase in IL-4 (51). Another meta-analysis found that sIL-2R and IL-12 increased and IL-1β, IL-2, and IL-6 decreased with antipsychotic treatment after a mean period of 53 days with antipsychotic treatment (2) including studies with both first-episode and chronic patients. The most consistent finding is a reduction in IL-2 and/or increase in sIL-2R. IL-2 is primarily secreted from activated T-lymphocytes, and is an immunoregulator stimulating growth and development of immune cells in peripheral tissue early in the immune response, and the growth of oligodendrocytes in neural tissue (28). Accordingly, the reduction of IL-2 after antipsychotic treatment implies a decreased immune response.

##### Deficit syndrome—negative symptoms

A study in patients with the deficit syndrome of schizophrenia—a subgroup of patients with primary negative symptoms from the illness debut—found significantly elevated IL-6 and TNF-α in patients with deficit syndrome compared to in non-deficit schizophrenia and healthy controls (52). The association between negative symptoms and elevated specific cytokines is particularly interesting as antipsychotics are not effective treatment options for negative symptoms. Drug trials with immunomodulating agents targeting negative symptoms are specificly warranted.

A recent meta-analysis by Goldsmith et al. (53) summarized existing findings regarding cytokine alterations in schizophrenia, bipolar disorder and major depressive disorder (MDD), and also compared results from acute and chronic phases. IL-6, TNF-α, IL-1RA, and sIL-2R were all elevated in the acute phases of all three disorders. After treatment, IL-6 decreased both in schizophrenia and MDD, while TNF-α did not change. In chronic states, IL-6 was elevated in all three disorders, while IL-1β and sIL-2R were elevated in schizophrenia and bipolar disorder. The authors conclude that there is a distinct similarity between the acute phases across all three diagnoses and they highlight that the cytokines with elevated levels in the three disorders are all modulated by nuclear factor-κB, regularly found to be activated in autoimmune and inflammatory disorders (53).

Some of the pro-inflammatory cytokines can barely be measured in healthy persons, while in infection-associated inflammatory responses, the concentrations rise 10 to 100-fold. In addition multiple confounders such as age, gender, smoking, body mass index (BMI), and diurnal variation may influence the results (2). Further, cytokine activity is inter-dependent with the hypothalamic pituitary adrenal axis. As a first psychotic episode generally induces high stress levels, it is unclear whether the observed cytokine rises are a general stress phenomenon, or a specific signature of psychosis. Finally, schizophrenia patients face a high burden of co-morbidity in terms of cardiometabolic disorders. As inflammation also plays a central role in the pathophysiology underlying these diseases, future research should address the nature of this relationship. Altered cytokine levels associated with schizophrenia could either be the result or cause of co-morbid disease, or there could be common immunopathogenetic mechanisms underlying both schizophrenia and for instance cardiovascular disease.

Having mentioned these problematic dimensions of cytokine measurement, the pro-inflammatory cytokines IL-1β, IL-6, and TNF-α seem to covariate with psychosis and could be useful tools for selecting participants to drug-studies with immune-modulating drugs targeting for example positive symptoms of schizophrenia.

#### Cytokine mRNA Levels

A study reporting differences in gene expression between 529 patients with schizophrenia and 660 healthy controls found 1,058 differentially expressed genes, of which 697 genes were upregulated (54). Gene set enrichment analysis showed that the upregulated genes were enriched in several processes involved in the response, activation and regulation of immunity. Differentially expressed immune genes included four complement genes: CR1, CR2, CD55, and C3, as well as TGFβ1 and TGM2. A study with combined measurement of mRNAs of cytokines and peripheral cytokines in plasma and serum aimed to define an inflammatory biotype of schizophrenia (20). This study used a recursive two-step cluster analysis to define subgroups of pro-inflammatory status on the basis of mRNAs of IL-18, IL-1β, IL-6, and IL-8. The cluster analysis included 68 controls and 82 patients. The results identified three clusters, cluster 1—low cytokine expression (*n* = 89), with below median expression of all measured cytokine mRNAs, cluster 2 (*n* = 50) was termed high cytokine expression and had above median for two and above third quartile for two cytokine mRNAs, while the very high cytokine expression group (*n* = 11) of cluster 3 were above the third quartile for all four cytokine mRNAs. 47.6% in the schizophrenia group was either cluster two or three compared to 32.4% in the healthy control group. The elevated/non-elevated subgroups of the schizophrenia participants did not differ with respect to gender, BMI, duration of illness or symptom severity as measured by the PANSS. Interestingly, the authors discussed the limited correlation between peripheral cytokine proteins and their mRNAs, and suggested that the main source of cytokine proteins may not be peripheral leucocytes. Furthermore, they suggested that mRNAs of pro-inflammatory cytokines could be used to select the patients who have an “elevated inflammation biotype” (20). In a study comparing 53 patients with schizophrenia and 53 healthy controls intracellular levels of IL-6 mRNA in the peripheral blood mononuclear cells (PBMC) analyzed with quantitative real-time polymerase chain reaction (RT-PCR) was found to be significantly elevated for patients with schizophrenia (55), and PBMC IL-6 mRNA was specifically suggested to be a candidate for a diagnostic marker for schizophrenia (55).

#### Acute Phase Proteins

CRP is an acute phase protein produced in the liver, stimulated by IL-1β, IL-6, and TNF (56) and released by macrophages and adipocytes. As demonstrated in the 2013 Guideline on the Assessment of Cardiovascular Risk from the American College of Cardiology and the American Heart Association (57) where CRP is now recommended as a supplementary test using a threshold of CRP ≥ 2 to indicate increased cardiovascular risk, CRP is widely used in clinical practice as a marker of inflammation. Another major advantage of CRP is that it can be measured reliably in most certified laboratories. A recent meta-analysis of 18 studies with 1,963 patients and 3,683 non-schizophrenia controls found that a diagnosis of schizophrenia was associated with a moderate increase in blood CRP (58), corroborating the results of a prior meta-analysis (4). Furthermore, patients from Asia or Africa and those who were younger than 30 years had higher CRP levels. The increase in CRP correlated with positive symptoms of schizophrenia but was unresponsive to initiation of antipsychotic treatment (4). A large and recent study (*n* > 1,000) reported elevated levels of CRP in patients with schizophrenia compared to controls, with levels of CRP correlating both to positive and negative symptoms (59). There is also evidence indicating a relationship between CRP and cognitive dysfunction in subjects with psychosis (60). In a recent systematic review by Orsolini et al. (61) elevated CRP levels were again identified in patients with schizophrenia and correlating with severity of symptoms. Interestingly, a large genome wide association study (GWAS) using mendelian randomization found that genetic factors that elevate CRP have a preventive effect with respect to developing schizophrenia (62), and the authors discussed that increased CRP in schizophrenia is more likely a result of developing and having the disease than being a predisposing factor. Various psychiatric disorders were investigated in a study assessing CRP in 599 admissions in a psychiatric catchment area. The prevalence of inflammation defined as CRP > 3 mg/L was 32% for psychotic disorders (ICD F 20–29), 21% for mood disorders (F30–39), 22% for neurotic disorders (F 40–48), and 42% for personality disorders (F60–69), indicating that low grade inflammation could be present in a whole range of psychiatric disorders (21). As obesity is more common among patients with a psychiatric diagnosis, the increase in adipose tissue and resulting higher risk for diabetes type 2 and cardiovascular illness could be an intermediating factor (63). Yet, even after adjusting for BMI CRP levels remain higher in patients (64). Furthermore, in a study of patients with first admissions to hospital with diagnoses of schizophrenia, bipolar disorders or depression, survival-analyses showed that moderately elevated CRP (3–10 mg/L) was associated with an increase in all-cause mortality with adjusted hazard rate (HR) of 1.56 (95% CI: 1.02–2.38), and for levels above 10 mg/L the adjusted HR was 2.07 (95% CI: 1.30–3.29). To conclude, the acute phase protein CRP is elevated in a proportion of individuals with schizophrenia and other psychoses, the measure is reliable and widely available, and it has been found to correlate both with positive symptoms of schizophrenia and cognitive function. Expression profiles of the additional acute phase proteins—haptoglobin (HP), alpha-1 antitrypsin (A1T), and alpha-2 macroglobulin (A2M) were investigated with quantitative polymerase chain reaction (qPCR) in a sample with 43 FEP patients and 57 healthy controls followed up for 3 months (65). All three acute phase proteins were elevated during the study period, and correlated with PANSS positive, depressive, and excitement subscales. The results are in line with previous studies using proteomic techniques identifying changes in acute phase proteins in patients with schizophrenia supporting that inflammation is an important feature in schizophrenia (66, 67).

#### Additional Circulating Proteins and Metabolites Related to Inflammation

Several methods with the capacity to identify and quantify several hundreds to thousand molecules simultaneously have been used to analyse blood sera from patients with schizophrenia. These techniques are referred to as proteomics using multiplex immunoassay, two-dimensional gel electrophoresis and mass spectrometry for identifying proteins, and metabolomics using metabolomics mass spectrometry and ^1^H-nuclear magnetic resonance spectroscopy (MRS) for identifying smaller circulating metabolites (68). Using proteomics, one interesting study comparing 17 drug-naïve FES to 17 healthy controls found that 9 proteins (creatine kinase m/B, MMP3, ACE, cortisol, TBG, α-2 macroblobulin, thrombopoietin, TSH, and ICAM-1) displayed lower concentrations in the patients vs. the controls (69). The authors commented that most of these proteins are involved in endothelial cell function and inflammation. Another study recently reported results from a novel proteomic method on PBMC from 20 patients with schizophrenia assessed both while acutely ill and in the recovery phase and compared to healthy controls. Interestingly, the study found significant differences in α-defencins 1–3 between the acutely admitted patients and healthy controls (70). A systematic review of metabolite biomarkers of schizophrenia that included 63 studies discussed their findings of decreased levels of the antioxidant vitamin E, polyunsaturated fatty acids (PUFAs), and phospholipids together with high levels of lipid peroxidation metabolites to indicate an oxidative balance favoring pro-oxidants and thus also inflammation in patients with schizophrenia (71). Although proteomics or metabolomics have not yet been applied as tools in treatment guidance for individual patients with schizophrenia, some have suggested how the use of these methods could improve treatment (11, 72). As recently proposed for mood disorders, using immune-based biomarkers together with traditional clinical descriptions of the individual patients may potentially improve both drug studies and individual treatment (73). Schwarz et al. (74) used proteomics to divide patients with schizophrenia into three groups: those with immune signature, those with growth factor disturbances and those with hormonal abnormalities. Such subdivisions could help to identify patient groups for specific augmentation therapy, for example with components such as NSAIDs, metformin or selective estrogen receptor modulators. Future drug trials should implement the promising results from this rapidly developing field in order to enable and provide time-efficient and personalized treatment options approaches. By combining several proteomic/metabolomic markers indicating inflammation in patients with schizophrenia, these methods could offer specific and sensitive ways to select participants for drug trials and monitoring drug effects on the molecular level (72).

##### Antibodies

Elevation of some antibody-titers has been linked to schizophrenia. A systematic quantitative review including 81 studies, showed that increased anticardiolipin IgG and N-methyl-D-aspartate (NMDA) receptor autoantibody titers and several additional autoantibodies were more prevalent in patients with schizophrenia (75). In contrast, another study of three cohorts of patients with schizophrenia stated that peripheral NMDA receptor autoantibodies are very rare in patients with schizophrenia (76). Also antibodies to gliadin have been found elevated in studies comparing patients with schizophrenia to healthy controls, but this was not the case for antibodies more specific to coeliac disease (77). While the recognition and early treatment of auto-immune encephalitis is an important part in the differential diagnosis of schizophrenia, it is unclear if the presence of auto-antibodies in serum without specific symptoms of auto-immune encephalitis (convulsions, rapid progression, decreased consciousness, and stereotypic movements) warrant additional treatment (78). Although of considerable interest, the origin and effect of these findings in patients with schizophrenia are not yet clear, and their usefulness in the mapping of inflammation in schizophrenia is elusive.

### Blood Brain Barrier Hyperpermeability

Evidence indicate increased permeability in the blood brain barrier (BBB) in a subset of patients with schizophrenia (79). One study found that 14 out of 39 patients with schizophrenia spectrum disorders displayed signs of BBB hyperpermeability including 9 patients with increased albumin cerebrospinal fluid (CSF)/serum concentration quotient (80). A recent meta-analysis by Orlovska-Waast et al. (81) with 32 studies concluded that patients with bipolar disorder and schizophrenia may display BBB abnormalities, but the authors also noted that the quality of available studies is rather low. Increased levels of S100B protein in blood and CSF that can be caused by BBB hyperpermeability (82) and elevated vascular endothelial growth factor (VEGF), a protein known to increase BBB permeability, have been found in patients with schizophrenia (83). Also increased levels of vascular endothelial adhesion molecules and integrin receptor have been detected in schizophrenia (84). A PET study focusing on P-glycoprotein (P-gp), a major efflux pump in the BBB, found it to be more active in schizophrenia (85) but this finding needs to be replicated. Increased BBB permeability can have deleterious effects on the brain by pro-inflammatory cells and molecules entering in brain (79). Although, there is no concensus regarding the best way to monitor increased BBB permeability in patients with schizophrenia, several novel candidates including matrix metalloproteinase-9(MMP-9), ubiquitin carboxy-terminal hydrolase-L1(UCHL-L1), neurofilaments, brain derived neurotropic factor (BDNF), miRNA in addition to S100B and glial fibrillary acidic protein (GFAP) are available for future studies, and preferably aggregated and applied in panels of several biomarkers (86).

### Assessments in the Brain

#### Neuroinflammation and Positron Emission Tomography (PET)

The concept of neuroinflammation indicates innate immune responses in the central nervous system (CNS) mainly produced by microglia and astrocytes (87), in contrast to the term neuroimmunology that denotes adaptive immunological changes within the CNS (88). However, the use of the term neuroinflammation to describe low-grade changes associated with depression and schizophrenia is controversial. One study that examined gene expression in brain in well-established inflammatory diseases (inflammatory bowel disease, juvenile dermatomyositis, MS, and ulcerative colitis) compared to the neurodevelopmental/neurodegenerative diseases Alzheimers disease, Parkinsons disease and schizophrenia reported a categorical difference between the neurodevelopmental/neurodegenerative diseases and the inflammatory diseases (89). The authors state that a distinction between classical neuroinflammatory conditions such as MS with typical mononuclear infiltrates and the smaller and ill-defined glial changes associated with secretion of various immune molecules must be established, and that missing this point may lead to unwarranted treatment trials (89). Nevertheless, classical neuroinflammation or more precisely microglial activation has been demonstrated to correlate with microglial expression of the translocator protein (TSPO) which is located at the outer mitochondrial membrane (90). While early studies with first generation TSPO PET tracers, for example [11C](R)-(1-[2-chrorophynyl]-N-methyl-N-[1-methylpropyl]-3 isoquinoline carboxamide (11C-(R)-PK11195) showed increased TSPO binding (91, 92), more recent studies did not find signs of microglial activation using the PK11195 tracer (93), and the specificity of TSPO binding to assess the inflammation associated with schizophrenia has been challenged (94). Second generation TSPO tracers have been developed, and in some studies increased microglial TSPO expression between patients with schizophrenia and healthy controls have been observed (95), while other did not find signs of increased TSPO expression (96). A recent meta-analysis that reviewed five studies with 75 patients and 77 healthy controls found that patients with schizophrenia had lower TSPO binding compared to controls, and concluded that this difference is caused either by lower density or altered function of brain immune cells (97). Hence, the advantage of measuring TSPO binding to assess low-grade inflammatory changes associated with schizophrenia seems to be in question, and the usefulness of the method to select patients with an “inflammatory” phenotype and monitoring effect of anti-inflammatory drugs appears to be low.

#### Magnetic Resonance Imaging (MRI) and Magnetic Resonance Spectroscopy (MRS)

MRI and MRS have also been proposed as potential means to measure the low-grade inflammatory changes associated with schizophrenia (98). MRS can measure concentrations of various molecules in defined volumes of the brain. Increased levels of glial markers as myo-inositol (MI), cholin (Cho) and total creatin, and reduced levels of neuronal markers as N-acetylaspartate and glutamate have been interpreted to indicate various dimensions of inflammation, such as increased density of glial cells and migration of glial cells into the inflamed area (98). MRS has so far been used in few studies to assess inflammation in schizophrenia. One study scanned 60 drug-naïve FES patients and 60 controls. The results showed elevated MI, Cho, and glutamate in the FES group (99) and were presented as evidence of inflammation in the early phase of schizophrenia. Also results from T1- and T2- weighted structural MRI and diffusion MRI free-water imaging may be used to assess low-scale brain inflammation (98, 100). The free-water imaging measures the amount of extracellular water in brain tissue and is postulated to correlate with oedema and possibly inflammation, and a few studies have identified increased free-water in samples of patients with schizophrenia compared to controls (101, 102). Taken together, MR based tecniques pose exciting opportunities to assess the subtle brain changes associated with schizophrenia. However, more research is needed to understand the relationship between these changes and other measures of inflammation, for example cytokine levels in CSF and the peripheral circulation, before they can be applied as markers of inflammation in the individual patient with schizophrenia. Prasad et al. found associations between diffusion tensor imaging measures and the levels of IL6 and CRP (103), but we have not identified studies exploring the relationship between more novel MRI methods and markers of inflammation in CSF or peripheral blood.

#### Cytokines in the Cerebrospinal Fluid

A meta-analysis including 16 studies comparing schizophrenia with healthy controls, found increased CSF levels of IL-1β, IL-6, IL-8, kynurenine, and kynurenic acid, while sIL-2R was decreased (104). With the exception of sIL-2R, increased levels of the same cytokines (IL-1β, IL-6, IL-8) have been reported in the periphery (2). A recent meta-analysis that included 32 studies also found that IL-6 and IL-8 were significantly elevated in schizophrenia (81). Presently it is not obvious that assessment of markers of inflammation in CSF to diagnose an inflammatory biotype in individual patients with schizophrenia will give additional gain vs. assessments in peripheral blood. With the exception of some countries, for example Germany and Denmark that investigate liquor in first episode patients to screen for Lyme's disease and other infectious causes, CSF assessment is not part of clinical practice for patients with psychosis. On the other hand, studies investigating CSF in schizophrenia are scarce and the number of participants are small compared to studies using peripheral measurements and future research might prove measuring inflammatory markers in CSF to be useful.

## Drug Studies to Treat Immune Dysregulation or Inflammation in Schizophrenia

### Non-steroidal Anti-inflammatory Drugs (NSAIDS)

Anti-inflammatory drugs have been used as add-on to antipsychotic treatment in patients with schizophrenia, with some success–see Table 1. The results are summarized in several meta-analyses: Sommer et al. included 4 studies with celecoxib, one with acetylsalicylic acid/aspirin (117), and reviewed 5 studies with celecoxib and 2 with aspirin in their broader meta-analysis of anti-inflammatory agents (16). A significant beneficial effect on PANSS total score of treatment with aspirin was found. As already mentioned, one randomized controlled trial (RCT) with aspirin showed stronger effects when stratifying participants on the basis of a marker of inflammation (27). Nitta et al. included 7 studies with celecoxib and 2 with aspirin (105). NSAIDS significantly reduced PANSS positive symptoms. The effects were moderated by aspirin treatment, in-patient treatment, first episode patients and lower PANSS negative scores. Zheng et al. conducted a meta-analysis including 8 studies with celecoxib conducted between 2002 and 2010, most of them were also included in the previously published meta-analyses (106). They concluded that treatment with celecoxib outperformed placebo in studies of first-episode patients, but not in patients with chronic schizophrenia. Effects were moderated by higher positive symptoms and lower negative symptoms at baseline. A few RCTs with anti-inflammatory drugs as add-on to antipsychotic treatment have been published after these overviews. Weiser et al. published a conference paper where they reanalyzed an RCT where patients with schizophrenia were randomized to treatment with either aspirine, minocycline, the dopamine-agonist pramipexole, or placebo (118). After subgrouping the participants according to CRP levels, they showed that in the upper third group with CRP above 3.8 mg/L, aspirine had a significant effect on PANSS positive symptoms. However, this was not found for other symptom measures, and not for participants treated with minocycline or pramipexole.

**Table 1:** Drug treatment of inflammation and immune dysregulation in schizophrenia.

**Drugs**	**Level of evidence**	**Effect on primary outcome**	**Effect on secondary outcome**
**ANTI-INFLAMMATORY DRUGS**			
**NSAIDs**			
Aspirin/acetyl**-**salicylic acid	Meta-analyses (16, 105)	↓PANSS total (16)	No effects in cognitive tests^c^
Celecoxib	Meta-analyses (16, 106)	↓PANSS total in subgroup first-episode patients (106)	↓Positive symptoms, negative symptoms, general psychopathology (106)
**Steroids**			
Prednisolone	Protocols for RCTs published^a^		
**NOVEL BIOLOGICALS—MONOCLONAL ANTIBODIES AGAINST CYTOKINES/CYTOKINE RECEPTORS/CELL ADHESION MOLECULES**			
Tocilizumab	One RCT in patients with residual symptoms (17)	No effect at week 12 on the PANSS total	No effects on the MATRICS, PANSS subscales, CGI, GAF, ↓CRP, ↑IL-6, and IL-8
	One open study (107)	↑BACS. No significant changes in psychopathology scores, cytokines or CRP	
	Protocol for RCT published^a^		
Siltuximab	Protocol for RCT published^a^		
Natalizumab	Protocol for RCT published^a^		
**MISCELLANEOUS DRUGS WITH ANTI-INFLAMMATORY ACTION**^**b**^			
N-acetylcysteine	Meta-analyses (16, 108)	↓Total psychopathology (SMD = −0.74). No effect on PANSS subscales (108)	No difference in any cause discontinuation rate or adverse drug reactions
Erythropoetin	One RCT^d^ (109)	↑RBANS subtests delayed memory, language–semantic fluency, attention. ↓WCST-64–perseverative errors	No effect on other cognitive tests, psychopathology, or MRI brain volumes
Pioglitazone	One RCT (110)	↓PANSS negative	↓PANSS total. No difference in PANSS positive, HDRS
Simvastatin	One RCT (111)	↓PANSS negative	↓PANSS total. No difference PANSS positive or general psychopathology
Pravastatin	One RCT (112)	↓LDL cholesterol. No effect on CRP, PANSS total score, MATRICS over 12 weeks	↓PANSS positive at 6 weeks
Minocycline	Meta-analyses (113, 114)	↓PANSS total (113, 114)	↓PANSS positive (113), negative (113, 114), and general psychopathology (113, 114) subscales
	Two recent RCTs (115, 116)	No effect on PANSS total (115) or PANSS negative subscale (116)	No effects on secondary outcomes: PANSS subscales, general psychopathology measures, cognition

a *At clinicaltrials.gov*.

b *Negative trials or drugs with negative findings in updated meta-analyses are not included*.

c *Assessed only in one study (27)*.

d *The inclusion criteria included an RBANS score < 1 SD below mean*.

*NSAIDS, non-steroidal anti-inflammatory drugs; PANSS, Positive and Negative Syndrome Scale; RCT, randomized controlled trial; CGI, Clinical global impression; GAF, Global assessment of functioning; CRP, C-reactive protein; IL, interleukin; BACS, The Brief Assessment of Cognition in Schizophrenia; SMD, standardized mean difference; RBANS, Repeatable Battery for the Assessment of Neuropsychological Status; WCST, Wisconsin Card Sorting Test; MRI, Magnetic resonance imaging; LDL, low density lipoprotein*.

### Steroidal Anti-inflammatory Drugs

Prednisolone, a synthetic corticosteroid, has been used for several decades in the treatment of various inflammatory and autoimmune disorders (119). The agent is more potent than NSAIDS as it targets several aspects of the immune system and interferes with almost all types of immune cells (120). Furthermore, prednisolone readily crosses the BBB. There are currently two recruiting randomized, placebo-controlled add-on trials investigating prednisolone in early phase schizophrena and related disorders (ClinicalTrials.gov Identifiers NCT03340909 and NCT02949232).

### Novel Biological Drugs With Immune-Modulating Actions

The availability of monoclonal antibodies toward cytokines, cytokine receptors or other specific parts of the immune system, in combination with findings of elevated cytokine levels in schizophrenia imply that studies with these drugs would strongly enhance our knowledge about cytokine dysfunction in schizophrenia. Drawing upon the experience with targeted immune therapy in diseases such as rheumatoid arthritis, psoriasis, Chrohns disease, ulcerative colitis, spondyloarthritis, and systemic lupus erythematosus, it would have been interesting to try out this strategy in patients with schizophrenia. However, caution is warranted, as the cytokine antagonists have a range of adverse effects due to the multiple and pleiotropic functions of the cytokines (121). According to Baker and Isaacs (18), the novel biological drugs with immune-modulating properties can be described according to their target of action: (1) drugs directed toward activity in the T-helper 17 immune axis: drugs directed toward IL-23p19, IL-17, or IL-12/23p40, (2) drugs active against type I and II Interferons, (3) drugs interfering in the lymphocyte recruitment by intervening in the adhesion process either by sphingosine−1-phosphate receptor inhibition or by integrin blockade, (4) Janus kinase inhibitors—first generation and second generation selective inhibitors, (5) drugs targeting B cells, (6) drugs modulating T cell function, and (7) bispecific antibodies.

Two studies on the effect of novel biologicals in patients with schizophrenia have been published, both with tocilizumab which is a humanized IL-6 receptor monoclonal antibody. Tocilizumab was tried in an open label study (107), and 2017 an RCT of 36 patients with residual symptoms of schizophrenia was published (17). In the study by Miller et al. (107), 6 participants (4 with schizophrenia and 2 with schizoaffective disorder) entered and 5 completed an 8 week open trial of adjunctive injections with 4 mg/kg tocilizumab at baseline and after 4 weeks, with the aim of studying the effect on cognition. All subjects improved in a processing speed measure, and 5 out of 6 improved also on a global cognition measure. The authors state that the signal-to-noise ratio may be increased in future studies if only patients with baseline inflammation were included, which was not the case in this study. Girgis et al. (17) reported a study where 36 clinically stable individuals with schizophrenia and PANSS total scores >60 were randomized to treatment with 3 monthly injections with 8 mg/kg tocilizumab or placebo at baseline and after 4 weeks. The results showed no effect of tocilizumab on any behavioral outcome, while CRP decreased and IL-6 and IL-8 increased. No prediction of outcome was seen by baseline CRP or the measured cytokines. No selection procedure based on elevated baseline inflammation measures was implemented, and the authors underlined the possibility that enriching the sample with inclusion of only individuals with an elevated CRP could have influenced the results.

We have not been able to identify other completed studies with novel biologicals targeting cytokines or cytokine receptors in schizophrenia. However, at the clinicaltrials.gov website three trials currently recruiting participants are listed, two from Augusta and one in London. One study conducted by the group of Brian Miller at Augusta University in Georgia, plans to randomize 30 stable outpatients with schizophrenia or schizoaffective disorder with CRP >5 mg/L to adjunct treatment with siltuximab which is a recombinant IL-6 monoclonal antibody, or placebo. In another study by the same group 20 stable outpatients with CRP >5 mg/L will be randomized to receive treatment with tocilizumab or placebo for 12 weeks. Lastly, a study conducted by Tiago Marques and Oliver Howes, Institute of Psychiatry at Kings college in London, plans to randomize 60 individuals with FES or other psychotic disorder to treatment with natalizumab, which is a humanized monoclonal antibody against the cell adhesion molecule α4-integrin, or placebo. The results are integrated in Table 1.

### Miscellaneous Drugs With Anti-inflammatory Actions

N-acetylcysteine (NAC) is a drug with several established indications, including the treatment of paracetamol intoxication. NAC interacts with a wide range of physiological pathways, and has anti-oxidative and anti-inflammatory effects (122). A recent systematic review and meta-analysis summarizing the results from 3 RCTs with schizophrenia patients found that NAC improved total psychopathology (108). A recent RCT including 63 early psychosis patients found that NAC had no effects on positive and negative symptoms or functional outcome, but a significant effect on processing speed (123). Interestingly, patients with higher values of glutathione peroxidase activity in blood cells (GPx_BC_), an indicator of oxidative stress and associated with brain glutathione levels, improved significantly on positive symptoms, leading the authors to suggest that GPx_BC_, could be a theranostic marker for NAC treatment (123).

Erythropoetin (EPO) is a glycoprotein secreted by the kidney in response to hypoxia that stimulates bone-marrow erythropoiesis. EPO also has an anti-inflammatory effect in the brain by modulating microglia responses and decreasing BBB hyperpermeability (124), among a wide range of other pharmacological actions. Recombinant EPO is used as a drug to treat anemia associated with kidney failure and cancer therapy, and has also been tried to improve cognition in patients with schizophrenia. A systematic review with a quantitative synthesis included 78 animal and human studies (125), including only one double-blind RCT (109). The results of this 3 month study showed that the patients with schizophrenia improved on all cognitive tests, but no improvement on the PANSS scores was found, and the authors suggest a role for EPO as an add-on treatment to antipsychotic drugs for patients with cognitive dysfunction. However, serious concerns regarding for example vascular side effects may limit the clinical use of EPO (125).

Statins have been tried in RCTs as add-ons to treatment with antipsychotics for patients with schizophrenia. Simvastatin showed effect on negative symptoms in an RCT with 66 patients randomized to simvastatin vs. placebo (111). According to the registrations on clinicaltrials (https://clinicaltrials.gov/), there are at least two on-going studies with simvastatin, one also with a published protocol (126). Vincenzi et al. randomized 60 patients with schizophrenia or schizoaffective disorder to pravastatine or placebo (112). Pravastatine significantly reduced PANSS total score from baseline to 6 weeks, but not at 12 weeks. Cholesterol and low density lipoprotein were reduced. In the subgroup with CRP above 2 mg/L cognitive measures were improved between baseline and 6 weeks.

Pioglitazone is an anti-diabetic drug also demonstrated to inhibit inflammatory pathways (127). Iranpour et al. randomized 40 patients with negative symptoms to pioglitazone vs. placebo as add-on to risperidone treatment and found significant decrease in negative symptoms after 8 weeks treatment (110).

Estrogens also have anti-inflammatory actions (128). The meta-analysis by Sommer et al. (16) included 8 studies, and concluded that treatment with estrogens have a significant effect on the PANSS total score. The same meta-analysis also included studies with davunetide (*n* = 2), fatty acids EPA/DHA(*n* = 7), and minocycline(*n* = 4). However, noen of these drugs showed a significant effect on the PANSS total score.

Minocycline is a second generation tetracycline with anti-inflammatory properties (129) and a potential for neuroprotective use for example after stroke (130). It has been tried for improving symptoms in schizophrenia in several studies, and the results summarized in meta-analyses (16, 113, 114). Solmi et al. (114) performed a systematic review and meta-analysis of studies published up to February 2016 and identified six RCTs with 215 participants randomized to minocycline and 198 to placebo. Minocyline was superior to placebo for PANSS total score, and the negative and general PANSS subscale, the conclusion was in contrast to the earlier meta-analysis by Sommer et al. (16) that included three studies with high heterogeneity. Xiang et al. (113) published a meta-analysis summarizing published RCTs upto January 2016 and included eight RCTs with 286 participants on minocycline and 262 on placebo. Also in this meta-analysis minocycline was superior to placebo in improving PANSS total, negative and general subscale, and here also for the positive subscale. However, two new RCTs do not find an effect of minocycline vs. placebo (115, 116). Deakin et al. (116) randomized 207 people with schizophrenia-spectrum disorder with an illness duration < 5 years to minocycline or placebo, half and half. No effect on the primary outcome PANSS negative subscale or other symptoms of schizophrenia was identified after 12 months treatment. Weiser et al. (115) randomized 200 people with schizophrenia or schizoaffective disorder to 16 weeks treatment with minocycline or placebo as add-on to antipsychotic treatment. No effects on the primary outcome PANSS total or the secondary outcomes PANSS subscales, Clinical Global Impression Scale–Severity or–improvement scales, Brief Assessment of Cognition in Schizophrenia and drop out rates were identified. Thus, after these two negative, but rather large and well performed RCTs the status of minocycline as an agent to treat patients with schizophrenia is rather cloudy. However, and related to the main aim of this paper, the trials did not enrich their sample for patients with elevated measures of inflammation. It can not be ruled out that the negative and contrasting findings in the minocycline literature are caused by applying the minocycline to an unselected sample (131). Also the results for miscellaneous drugs are summarized in Table 1.

### Theranostic Biomarkers in Studies With Anti-inflammatory/Immune-modulating Drugs for Patients With Schizophrenia

In summary, we did not find any completed studies on anti-inflammatory treatment or immune-modulating drugs that have selected patients based on elevated markers of inflammation. A few studies have analyzed subgroups of participants with for example elevated CRP, and indications of more beneficial effects of anti-inflammatory treatment in subgroups with raised CRP have been identified. However, this applies only to a minority of studies, and it is conceivable that the influence of anti-inflammatory treatment could have been stronger if the treatment were targeted to participants with elevated markers of inflammation. Several protocols for ongoing add-on trials have been published at clinicaltrials.gov/ and a few of these seem to plan selection of participants based on an elevated CRP level. If this strategy is successful, CRP could be regarded as a first theranostic biomarker for tailoring anti-inflammatory/immune-modulating treatment to patients with schizophrenia, which could pave a way for more sophisticated markers in the future. An interesting clustering of well-known and putative immune-modulating drugs based on the *in-vitro* influence on cytokine levels were done by Wallner et al. (132). They identified cyclosporine A and tacrolimus among other drugs as one group predominately influencing IFN-gamma, IL-2 and IL-17, while the JAK family of drugs inhibits IFN-γ and increases IL-2 and the chemokine MIP3-α. Consequently, if a specific immune/inflammation activation pattern can be repeatedly identified in patients with schizophrenia, a more specific and hopefully more effective immune-modulating drug therapy can be explored in future trials.

## Constructing the Inflammatory Signature of Schizophrenia for Clinical and Research Purpose

The search for theranostic biomarkers for anti-inflammatory or immune-modulating treatment is still in its infancy. When selectively reviewing how inflammation and immunological dysregulation can be assessed in order to diagnose low-grade inflammation in patients with schizophrenia, two main issues need to be addressed. Firstly, the research conducted so far regarding immune dysregulation in schizophrenia has been focused to a large extent on finding differences on a group level comparing patients with schizophrenia and healthy controls, rather than determining the level of inflammation in the individual patient. Many advanced methods and bioinformatical tools are increasingly available and will doubtless be further developed to uncover cell populations or molecules that could serve as theranostic biomarkers and help assess the chances that anti-inflammatory or immune-modulating treatment will be of help to the individual patient. Secondly, although studies with proteomics, metabolomics and brain imaging offer exciting possibilities, presently the less sophisticated option of measuring the protein level of CRP and pro-inflammatory cytokines—IL-1β, IL-6, TNF-α, TGF-β–in serum is far more convenient for identifying patients with low-grade inflammation. Among the pro-inflammatory cytokines, IL-6 is found most often to be elevated in patients with schizophrenia (3), and has also been suggested as a possible state marker for acute relapse (2). However, as IL-6 seems to be both pro- and anti-inflammatory, more research is needed to establish the usefulness of IL-6 as a biomarker in schizophrenia (39). The general problem of assaying cytokines reliably and standardized remains and constrains further development of the field. Furthermore, and as discussed by Goldsmith et al. (53), it would be useful to agree internationally on a panel of cytokine network components to be studied in order to increase comparability between studies, and also to evaluate patterns of cytokines for example with cytokine ratios instead of focusing on individual cytokines.

There is some evidence indicating a link between peripheral levels of inflammation and symptom severity. For instance, CRP levels correlates with positive symptoms, negative symptoms, and cognitive dysfunction (60) and a level of CRP >3.8 mg/L increased the chance of therapeutic response to anti-inflammatory treatment with aspirin in one study (118). Besides this unique study, the literature offers little guidance as to the use of inflammatory parameters as theranostic biomarkers. In the current protocols for two ongoing studies with monoclonal antibodies conducted by dr. Brian Miller at Augusta University, the CRP level of 5 mg/L is chosen. In another study with add-on prednisolone, sponsored by Erik Johnsen at the Unversity of Bergen, a CRP level of >3.8 mg/L is chosen as an inclusion criterion (clinicaltrials.gov), based on the study by Weiser et al. (118). Adding to this, and based on the *in-vitro* findings of Wallner et al. (132), specific drugs could probably use specific measures to show response of treatment. We are certain that drug trials with anti-inflammatory or immune-modulating drugs would benefit from an concenting scientific community on how to select participants. This would increase comparability and also the possibility to show effect. Another important aspect calling for international collaborations is the need to increase the number of participants in drug trials in order to explore the fascinating possibility of disease-modifying treatment for schizophrenia posed by immune-modulating drugs.

Moreover, it seems clear that inflammation in this context should be regarded more as a dimension rather than a category. Thus, determining the degree of inflammation that causes or contributes to increased symptoms or deteriorating function is essential in the development of novel theranostic biomarkers (133). This can only be done in large longitudinal studies where multiple markers are monitored and predictors for increased symptoms, decreased function and other adverse developments are identified. Currently, biomarker panels are preferred to single markers (86), and individual differences must be accounted for (11).

## Summary

- The existing knowledge does not provide evidence to conclude that all patients with schizophrenia have increased inflammation, on the contrary; most studies report a low-grade inflammation in a subset of around 35–50% of patients with schizophrenia, and in the transition from descriptives on a group level to individual diagnosis the best way to diagnose inflammation in schizophrenia must be defined
- CRP and the pro-inflammatory cytokines are the proteins that at present should be further validated as biomarkers for inflammation in schizophrenia. Furthermore, they correlate at least in some studies with symptoms and phases of illness, and measurement is standardized and available from clinical laboratories
- More methods have the potential to develop biomarkers for inflammation in schizophrenia: immune cell characteristics measured by flow cytometry, proteins measured by proteomic methods, or other molecular elements mapped by metabolomics.
- The ultimal goal would be a panel of molecules with proven specificity and sensitivity as theranostic biomarkers which could be supported by brain imaging methods—to select patients for anti-inflammatory or immune-modulating treatment. Large longitudinal studies following standard development for biomarkers are needed to fulfill this goal.

## Author Contributions

RK, IS, VS, and EJ drafted the study. RK did the literature searches and wrote the first version. IS, VS, ID, and EJ commented, finalized the paper, and all approved the final version.

### Conflict of Interest Statement

The authors declare that the research was conducted in the absence of any commercial or financial relationships that could be construed as a potential conflict of interest.
